# Improved Daily Spatial Precipitation Estimation by Merging Multi-Source Precipitation Data Based on the Geographically Weighted Regression Method: A Case Study of Taihu Lake Basin, China

**DOI:** 10.3390/ijerph192113866

**Published:** 2022-10-25

**Authors:** Yi Pan, Qiqi Yuan, Jinsong Ma, Lachun Wang

**Affiliations:** School of Geography and Ocean Sciences, Nanjing University, Nanjing 210023, China

**Keywords:** MSWEP, gauge observation, precipitation fusion, geographically weighted regression

## Abstract

Accurately estimating the spatial and temporal distribution of precipitation is crucial for hydrological modeling. However, precipitation products based on a single source have their advantages and disadvantages. How to effectively combine the advantages of different precipitation datasets has become an important topic in developing high-quality precipitation products internationally in recent years. This paper uses the measured precipitation data of Multi-Source Weighted-Ensemble Precipitation (MSWEP) and in situ rainfall observation in the Taihu Lake Basin, as well as the longitude, latitude, elevation, slope, aspect, surface roughness, distance to the coastline, and land use and land cover data, and adopts a two-step method to achieve precipitation fusion: (1) downscaling the MSWEP source precipitation field using the bilinear interpolation method and (2) using the geographically weighted regression (GWR) method and tri-cube function weighting method to achieve fusion. Considering geographical and human activities factors, the spatial and temporal distribution of precipitation errors in MSWEP is detected. The fusion of MSWEP and gauge observation precipitation is realized. The results show that the method in this paper significantly improves the spatial resolution and accuracy of precipitation data in the Taihu Lake Basin.

## 1. Introduction

Precipitation is an important part of the water cycle and energy exchange in the climate system. It is an important indicator to characterize climate change [1,2,3,4]. Floods, droughts, and other extreme precipitation weather adversely affect human production and life [5,6,7,8,9,10,11]. Therefore, accurate estimation of the temporal and spatial distribution of precipitation is crucial for understanding the climate system and long-term weather climate variability, as well as forecasting hydrological processes [12,13,14].

There are five main ways to obtain precipitation observation information: in situ rain gauges, ground-based radar, satellite remote sensing detection information, model forecast, and reanalysis of precipitation products [15,16,17]. Precipitation products based on a single source have advantages and disadvantages. On the one hand, the precipitation information from ground rain-gauges and ground-based radar is relatively accurate, but their spatial distribution is extremely nonuniform, which is suitable for observing local or regional precipitation, but it is particularly difficult to obtain more accurate large-scale or global precipitation information [15,18,19]. On the other hand, precipitation satellites can carry out continuous detection in a large range of space. The time resolution of some specific target areas is also very high, but the accuracy is relatively low [20,21,22,23]. Due to the lack of a comprehensive observation system with extensive spatiotemporal continuous coverage, it is very difficult to obtain a long-sequence, full coverage of the global precipitation distribution with high spatiotemporal resolution and high precision. Precipitation products from numerical models can make up for the problems caused by the discontinuity of the observation system in space and time, but model precipitation also has serious systematic deviations [16,17].

Therefore, how to effectively combine the advantages of precipitation information from different data sources and develop multi-source precipitation fusion technology to minimize hydrological response errors and improve surface model prediction skills has recently become a popular topic in developing high-quality precipitation products internationally [24,25,26,27,28]. For example, the Tropical Rainfall Measuring Mission (TRMM)_3B42 and Climate Prediction Center Morphing Technique (CMORPH) have been widely used, and these products effectively combine the advantages of rainfall data from different sources. The TRMM Multi-satellite Precipitation Analysis (TMPA) algorithm adopted by the National Center for Environmental Prediction (NCEP) first uses TRMM Microwave Imager (TMI) precipitation to correct the passive-microwave (PMW) precipitation of multiple polar-orbiting satellites and then infrared brightness temperature-based precipitation retrieval coefficients are established by matching samples of corrected PMW precipitation and geostationary satellite infrared radiation (IR) observations [29]. The morphing (CMORPH) technology of the National Oceanic and Atmospheric Administration (NOAA) Climate Prediction Center (CPC), which uses IR data to calculate the cloud movement vector, extrapolates the PMW precipitation forward and backward and obtains the global CMORPH precipitation product with a temporal resolution of 30 min and a spatial resolution of 8 km [30]. Additionally, the recently developed Multi-Source Weighted-Ensemble Precipitation (MSWEP) is a global precipitation dataset that incorporates not only information from rain gauges and satellites but also model reanalysis data, covering the entire Earth with a timespan from 1979 to 2022, with a 0.1° spatial resolution and 3-hours temporal resolution. The global and regional accuracy evaluation results show that MSWEP precipitation products’ accuracy is generally higher in the same type of fusion precipitation data, and it is better than the widely used TRMM_3B42 and CMORPH, especially in data-scarce areas [31]. However, with the deepening of related research on MSWEP data, the problem of low-quality surface reference data in the accuracy evaluation of MSWEP precipitation data has become increasingly prominent [32]. Therefore, to obtain more accurate regional precipitation information with a higher spatial and temporal resolution, it is necessary to perform spatial precipitation correction on MSWEP by integrating the precipitation measured by ground rain gauges.

The difficulty in fusing precipitation data from different sources lies in estimating the temporal and spatial distribution of precipitation product errors. The geographically weighted regression (GWR) method can remedy this. Geographically weighted regression models can explore the relationship between the response variable and a set of covariates at a certain scale for the research subject by establishing a local regression equation at each point in the spatial range. This model is suitable for the quantitative simulation of non-stationary spatial relationships between variables and is widely used in geography and other fields [33,34,35,36,37,38]. Some researchers have tried to characterize the precipitation error field through spatial variables; topographic variables; and meteorological variables, including longitude, latitude, and elevation [39,40]. At the same time, GWR technology has been continuously innovated and evolved and has continued to expand in terms of distance measurement, multi-scale estimation, and spatiotemporal synthesis. The wide application of GWR in various fields and the continuous development of technology also show that it has become one of the important modeling tools [41,42,43,44].

In this study, we aim to develop a method to fuse the precipitation measured by rainfall stations based on multi-source fusion precipitation MSWEP to correct the deviation in MSWEP and obtain higher-precision precipitation products. At the same time, the precipitation product is downscaled to a finer spatial resolution while maintaining accuracy. The precipitation products with improved accuracy and spatial resolution can be used for hydrological modeling and forecasting.

## 2. Materials and Methods

### 2.1. Study Area and Data

#### 2.1.1. Study Area

This study selected the Taihu Lake Basin in China as the study area. The basin is located in the midlatitude and covers an area of 36,900 square kilometers. The administrative division includes most of the southern part of Jiangsu Province, Huzhou and Jiaxing and Hangzhou in Zhejiang Province, and most of Shanghai. The Taihu Lake Basin is dominated by plains, accounting for 4/6 of the total area, with hills and mountains accounting for 1/6, and water surface accounting for 1/6. The Taihu Lake Basin belongs to the humid north subtropical climate zone, and the climate has obvious monsoon characteristics and four distinct seasons. In winter, there is cold air intrusion, mostly northerly winds, cold and dry; at the turn of spring and summer, warm and humid air flows northward, and cold and warm air currents encounter to form continuous overcast and rainy areas, called “meiyu”, which can cause flood disasters. Under the control of subtropical high pressure, the weather is sunny and hot in mid-summer. It is often affected by tropical storms and typhoons at this moment, resulting in severe weather with strong winds and heavy rain. The annual average temperature in the basin is 15 ℃–17 ℃, increasing from north to south. The annual average rainfall is 1181mm, of which 60% is concentrated in May–September. The interannual variation of precipitation is large, and the ratio of maximum to minimum annual precipitation is 2.4. However, the interannual variation of annual runoff is larger, and the ratio of maximum to minimum annual runoff is as high as 15.7. At the same time, the Taihu Lake Basin has superior natural conditions, convenient water and land transportation, good basic conditions for agricultural production, developed industry, strong economic foundation, dense population, high-quality labor force, strong scientific and technological strength, well-informed market information, and good infrastructure and investment environment. It is the main open area along the coast of China.

#### 2.1.2. Rain Gauge Data

**Figure 1:**
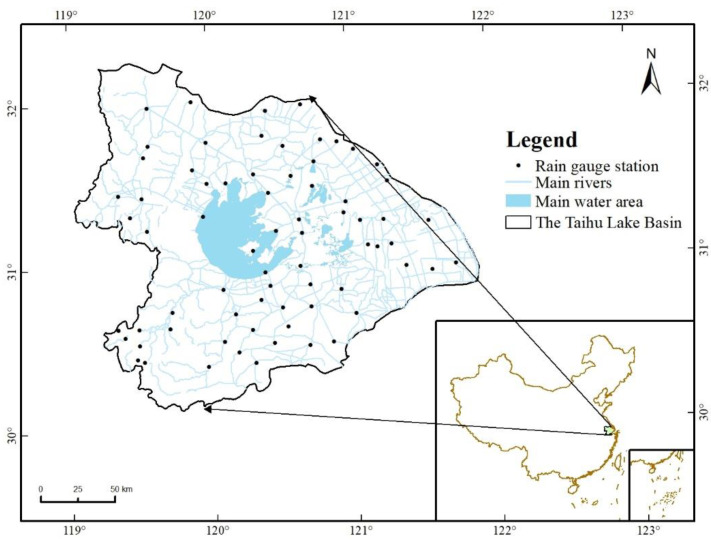
Map of the study area and location of the rain gauge stations.

The daily time series of the 70 rain gauges used in this study were mainly from the Basin Hydrological Yearbook, and all data were subject to strict quality control. A map of the study area and the rainfall stations are shown in Figure 1. On average, there is only one rainfall station per 527 square kilometers. The precipitation results obtained by directly interpolating the daily observational precipitation data of the rain gauge are poor. Multi-source precipitation fusion data are more representative. It is also necessary to carry out spatial precipitation correction on source precipitation products in combination with a large number of ground-measured precipitations to obtain spatially fused precipitation data that can better reflect the actual regional precipitation.

#### 2.1.3. The Source Precipitation Products

In this study, we selected the multi-source weighted-ensemble precipitation fusion data MSWEP as the source precipitation field. The product integrates site data, satellite observation data, and model simulation data and has a long time scale, large coverage, and high temporal and spatial resolution. MSWEP, a gridded product with global 0.1° spatial resolution and 3 hours temporal resolution, was used in this study as the precipitation background field to be corrected. In the study area of this paper, 0.1° is about 10.8 km along the latitude and about 9.2 km along the longitude. In this paper, MSWEP is preprocessed to reduce the spatial resolution to 1 km by bilinear interpolation and merge the temporal resolution to one day.

#### 2.1.4. Explanatory Variables

**Figure 2:**
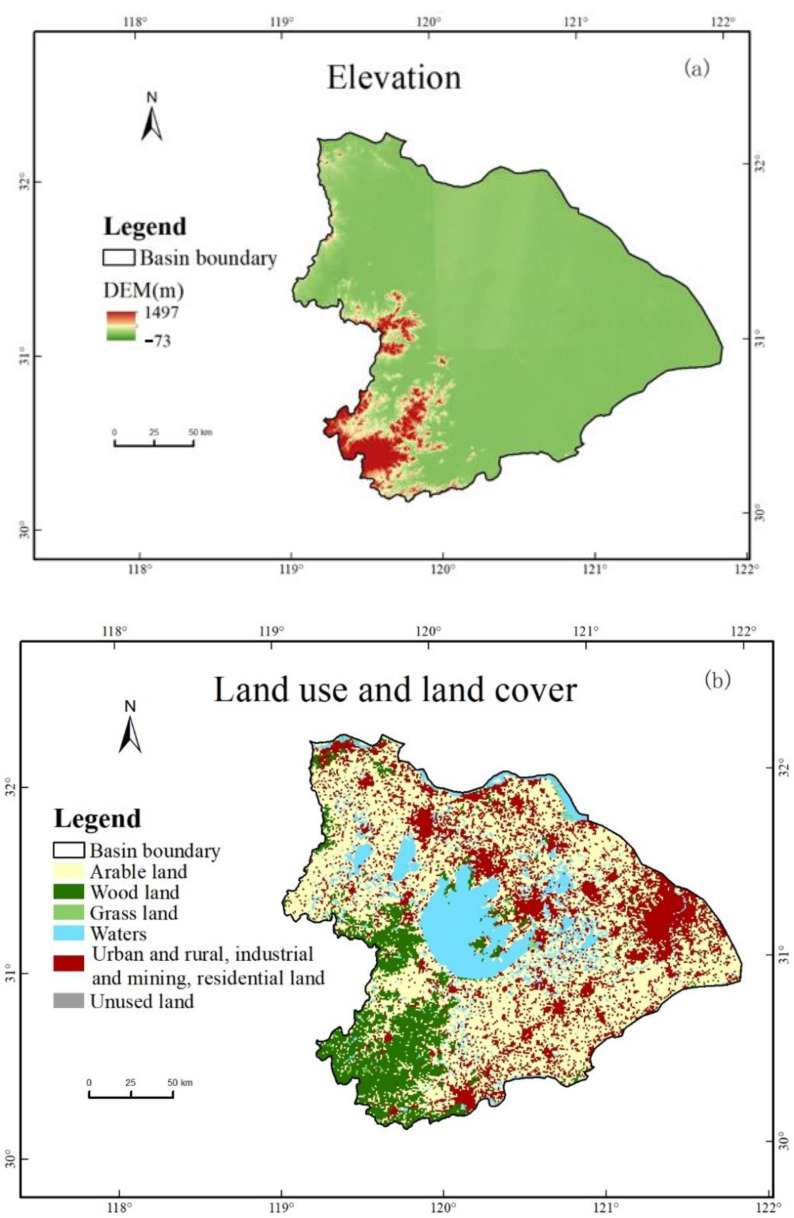
(**a**) Elevation and (**b**) land use and land cover for the Taihu Lake Basin.

In addition, this study used 30-m digital elevation map (DEM) data publicly available from the United States Geological Survey (USGS). The DEM data are aggregated from 30 m to 1 km, and data such as longitude, latitude, elevation, slope, aspect, surface roughness, and distance to the coastline are further extracted from the DEM data with a resolution of 1 km. Beyond that, this study also used land use and land cover (LULC) data with a spatial resolution of 1 km from the Resource and Environmental Data Cloud Platform (REDCP). Two types of data, DEM and LULC, are used to explore the possible effects of common geographic factors and human activities on precipitation. The elevation and land use and land cover of Taihu Lake Basin are shown in Figure 2.

### 2.2. Methods

#### 2.2.1. Bilinear Interpolation

**Figure 3:**
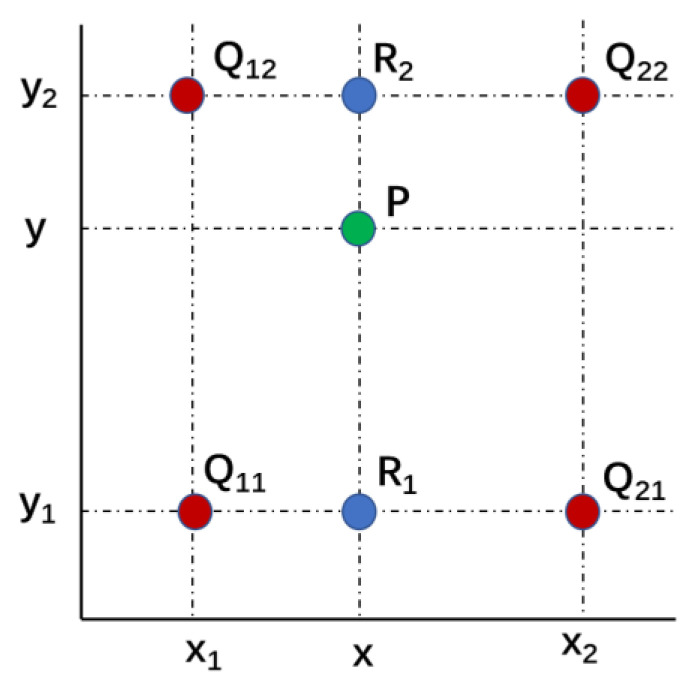
Principle diagram of bilinear interpolation.

There are many ways to perform interpolation techniques, and the most commonly used algorithms are nearest interpolation, bilinear interpolation, and bicubic interpolation. These three interpolation methods have advantages and disadvantages [45,46]. Nearest interpolation is one of the most basic and simple image scaling algorithms, which has the fastest computation speed but the worst effect [47]. Bicubic interpolation uses 16 (4 × 4) points in the original image to calculate 1 point in the new image. It works well, but it is a computationally expensive algorithm [48]. Bilinear interpolation uses 4 (2 × 2) points in the original image to calculate 1 point in the new image. The effect is slightly worse than bicubic interpolation, and the speed is faster than bicubic interpolation. It is a balanced algorithm that belongs to the default algorithm of many frameworks [49,50]. As an interpolation algorithm in numerical analysis, bilinear interpolation is widely used in signal processing and digital image and video processing. In this paper, it is applied to MSWEP to increase the data density and improve the spatial resolution of precipitation products. The main idea of bilinear interpolation is to perform linear interpolation in one direction and then operate it once more in the other direction. Whether you interpolate first on the *X* axis or the *Y* axis is the same, the order does not affect the interpolation result. The principle diagram of bilinear interpolation is shown in Figure 3.

In Figure 3, the values of the function *f* at the four points *Q*_11_, *Q*_12_, *Q*_21_, and *Q*_22_ are known, and bilinear interpolation is used to solve the value of the function *f* at the unknown point *P*. Most commonly, *f* is the grid value of a grid. Bilinear interpolation consists of two steps. First, linear interpolation is performed in the X direction:

(1) $$f\left(R_{1}\right)\approx \frac{x_{2}-x}{x_{2}-x_{1}}\;f\left(Q_{11}\right)+\frac{x-x_{1}}{x_{2}-x_{1}}\;f\left(Q_{21}\right)$$

(2) $$f\left(R_{2}\right)\approx \frac{x_{2}-x}{x_{2}-x_{1}}\;f\left(Q_{12}\right)+\frac{x-x_{1}}{x_{2}-x_{1}}\;f\left(Q_{22}\right)$$

where *Q*_11_ = (*x*_1_,*y*_1_), *Q*_12_ = (*x*_1_,*y*_2_), *Q*_21_ = (*x*_2_,*y*_1_), *Q*_22_ = (*x*_2_,*y*_2_), *R*_1_ = (*x*,*y*_1_), and *R*_2_ = (*x*,*y*_2_). *f*(*R*_1_) is the result of linear interpolation on the *X* axis between *Q*_11_ and *Q*_21_, and *f*(*R*_2_) is the result of linear interpolation on the *X* axis between *Q*_12_ and *Q*_22_.

Second, linear interpolation is performed in the *Y* direction:

(3) $$f\left(P\right)\approx \frac{y_{2\;}-y}{y_{2\;}-y}\;f\left(R_{1}\right)+\frac{y-y_{1}}{y_{2\;}-y_{1}}\;f\left(R_{2}\right)$$

where *P* = (*x*, *y*), *f*(*P*) is the result of linear interpolation between *R*_1_ and *R*_2_. According to Formulas (1)–(3), we obtain the value of the function *f* at the unknown point *P*.

#### 2.2.2. Geographically Weighted Regression

Geographically weighted regression methods can explore the relationship between the response variable and a set of covariates at a certain scale of the research object by establishing a local regression equation at each point in the spatial range. In recent years, it has been widely used in social economics, urban geography, meteorology, and other fields [33,34,35,36,37,38]. It has also attracted much attention in the application of precipitation fusion. In this paper, we attempt to construct a GWR model to explore the relationship between precipitation and related geographic elements and aim to obtain a more accurate spatial distribution of precipitation products by correcting the precipitation error field. The specific steps are as follows [51]:

(4) $$P_{F}=P_{M}+f(P_{O}-P_{M})$$

where $P_{F}$ is the final merged precipitation, $P_{M}$ is the MSWEP precipitation, and $P_{0}$ is the gauge observation precipitation. $f\left(P_{0}-P_{M}\right)$ represents the bias that needs to be corrected in the MSWEP precipitation. In this paper, first, the MSWEP precipitation product is used as the initial precipitation. Second, the precipitation error field of Taihu Lake Basin is solved by mathematical optimization method based on the error of the gauge observation precipitation. Third, the final merged precipitation is obtained by merging the MSWEP precipitation product and the error field. The basic idea of our research is consistent with the basic idea of most data fusion methods. The difference is that the GWR method is used to solve the error field of MSWEP precipitation in this paper.

In this paper, several geographical elements are selected as explanatory variables, and the error between MSWEP precipitation products and measured rainfall is taken as response variables, which is directly related to the six parameters of elevation, slope, aspect, surface roughness, distance to the coastline, and land use and land cover to construct a GWR model.

(5) $$f{\left(P_{O}-P_{M}\right)}_{i}=y_{i}=\beta_{i0}(\mu_{i},\nu_{i})+\sum_{k=1}^{p}\beta_{ik}(\mu_{i},\nu_{i})x_{ik}+\varepsilon_{i}$$

where $y_{i}$ is the estimated value of error at rainfall stations or grid pixels, $\left(\mu_{i},v_{i}\right)$ is the coordinates of rainfall stations or grid pixels, $\beta_{i0}$ is the constant regression coefficient at location i, and p is the number of explanatory variables here; there are 6: elevation, slope, aspect, surface roughness, distance to the coastline, and land use and land cover, $\beta_{ik}$ (k=1,2,3,…,p) are the regression parameters at location i, and $\varepsilon_{{}_{i}}$ is the regression residual at location i.

The regression coefficients of rainfall stations or grid pixels are estimated as follows:

(6) $$\hat{\beta }(\mu_{i},v_{i})=(X^{T}W_{i}(\mu_{i},\nu_{i})X{)}^{-1}X^{T}W_{i}(\mu_{i},\nu_{i})y$$

where $\hat{\beta }(\mu_{i},v_{i})$ is the vector of estimated coefficients at location i which is at rainfall stations or grid cells, X is the explanatory variable matrix, and y is the deviation of the measured precipitation value from the estimated value of the precipitation product. $W_{i}(\mu_{i},\nu_{i})$ is a diagonal matrix, and the diagonal elements are the spatial weights between position i and several adjacent rainfall stations.

(7) $$W_{i}(u_{i},v_{i})=\left[\begin{array}{cccc} w_{i1} & 0 & \begin{array}{ccc} . & . & . \end{array} & 0\\ 0 & w_{i2} & \begin{array}{ccc} . & . & . \end{array} & 0\\ \begin{array}{l} .\\ .\\ . \end{array} & & \begin{array}{ccc} . & & \\ & . & \\ & & . \end{array} & \begin{array}{l} .\\ .\\ . \end{array}\\ 0 & 0 & \begin{array}{ccc} . & . & . \end{array} & w_{in} \end{array}\right]$$

where $w_{ij}(j=1,2,\ldots ,n)$ is the weight value from the data point j to the regression analysis point i, which needs to be estimated using a weighting function [52]. For the solution of GWR model, commonly used weighting functions include Gaussian function (Equation (8)), exponential function (Equation (9)), bi-square function (Equation (10)), and tri-cube function (Equation (11)). According to the range distribution characteristics of kernel function, functions can be divided into continuous types and truncated types. Gaussian function and exponential function are continuous, while bi-square function and tri-cube function are truncated [53].

(8) $$w_{ij}=e^{-\frac{{}^{{\left(d_{ij}/b\right)}^{2}}}{2}}$$

(9) $$w_{ij}=\exp(-\frac{\left|d_{ij}\right|}{b})$$

(10) $$w_{ij}=\{\begin{array}{c} {\left(1-{\left(d_{ij}/b\right)}^{2}\right)}^{2},d_{ij}\leq b\\ 0,else \end{array}$$

(11) $$w_{ij}=\{\begin{array}{c} {\left(1-{\left(d_{ij}/b\right)}^{3}\right)}^{3},d_{ij}\leq b\\ 0,else \end{array}$$

where $d_{ij}$ is the distance between the position j and the position of the rain gauge station i, and b is the spatial bandwidth. The distance $d_{ij}$ is calculated by longitude and latitude data. The spatial bandwidth b is an important control parameter for the weight calculation of the GWR model, which directly determines the rate at which the weight decays with increasing distance or the range of valid data points around each regression analysis point during the GWR model’s solution process. Therefore, solving the appropriate bandwidth is a necessary procedure for solving the GWR model. Spatial bandwidth can be determined according to the Akaike information criterion (AIC) or derived from cross-validation residual sum of squares (CVRSS) [54,55]. In this paper, the corresponding bandwidth of minimum CVRSS is used as the optimal bandwidth to calculate the weight function.

(12) $$CV(b)=\sum_{i=1}^{n}(y_{i}-{\hat{y}}_{\neq_{i}}(b){)}^{2}$$

In Equation (12), ${\hat{y}}_{\neq_{i}}(b)$ refers to the estimation of parameters based on the removal point i.

**Figure 4:**
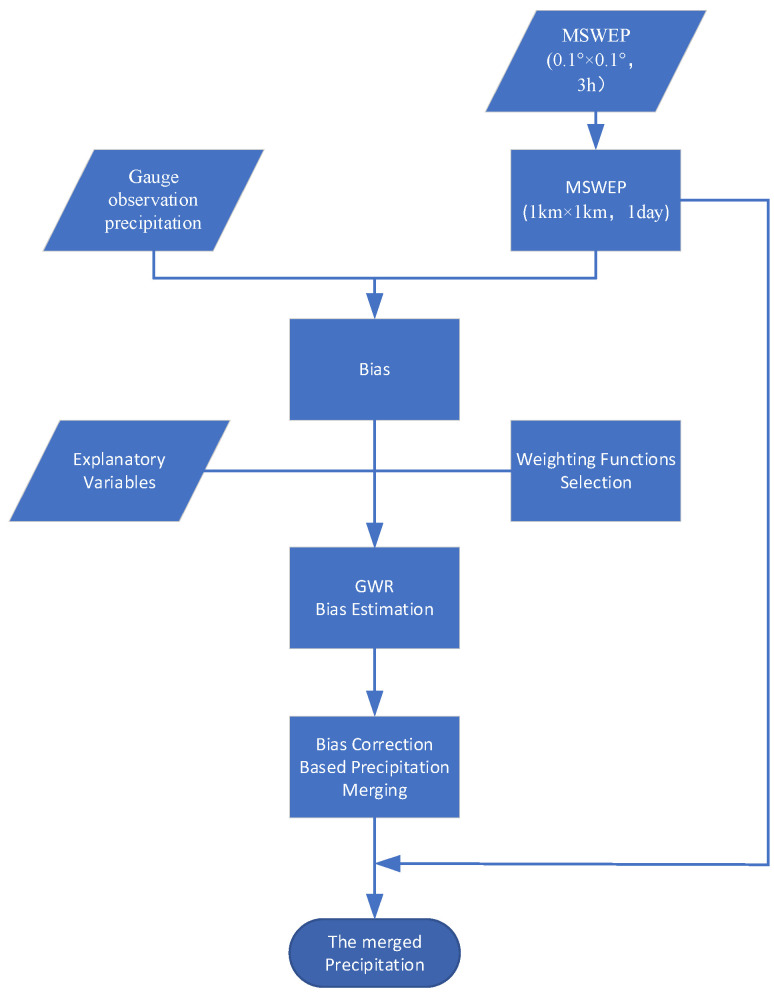
Flowchart of the GWR-based two-step merging scheme proposed in this study.

Finally, the flowchart of the GWR-based two-step merging scheme proposed in this study is shown in Figure 4.

### 2.3. Validation

In this paper, the cross-validation method leave-one-out cross-validation (Loo-CV) is used to evaluate the effectiveness of the precipitation data fusion method. The basic idea of Loo-CV is to select *m* − 1 samples from *m* samples to train the model, leaving one validation model, and repeat this step *m* times—that is, until all data points are used for validation. One advantage of this method is that all samples are used to train the model, so it has the closest distribution to the original samples.

Statistical metrics used to quantify the accuracy of merge algorithms include continuous indices and categorical indices. Continuous indices include correlation coefficient (CC), relative BIAS (rBIAS), root mean square difference (RMSD), and Kling–Gupta efficiency coefficient (KGE). The CC reflects the degree of fit between the estimated value and the observed value, with the best value being 1; the rBIAS describes the systematic deviation of the estimated value, and the best value is 0%; the RMSD visually shows the accuracy of the merged results, and the best value is 0. In addition, KGE is calculated to compare the observed value with the estimated value. KGE contains three components, namely, correlation coefficient, bias ratio, and variability ratio, and the optimal score is 1.

At the same time, categorical indices are also used to evaluate the accuracy of merge algorithms. In this paper, precipitation values are divided into five categories, including no rain ([0,1) mm/d), light rain ([1,5) mm/d), moderate rain ([5,20) mm/d), heavy rain ([20,40) mm/d), and violent rain (≥40 mm/d)), according to precipitation intensity, as well as four statistical classification indicators, including probability of detection (POD), false alarm ratio (FAR), frequency bias index (FBI), and critical success index (CSI), which are also calculated to quantitatively evaluate the precipitation detection ability of data from different sources. POD describes how often the merging product correctly detects precipitation events. FAR indicates the proportion of cases where the merging product recognizes rain when there is no rain measured by the rain gauge. FBI compares the number of events identified by the merging precipitation product to the number of events recorded by the rain gauge. When the FBI is greater than 1, the fused precipitation product overestimates the occurrence of the corresponding precipitation event, while a FBI less than 1 indicates underestimation. The CSI shows the overall ability of the fused precipitation product to correctly diagnose precipitation events. The optimal score of FAR is 0, and the optimal score of the other three classification statistical indicators is 1.

## 3. Results

### 3.1. Local Regression Analysis

#### 3.1.1. Sensitivity Analysis

**Figure 5:**
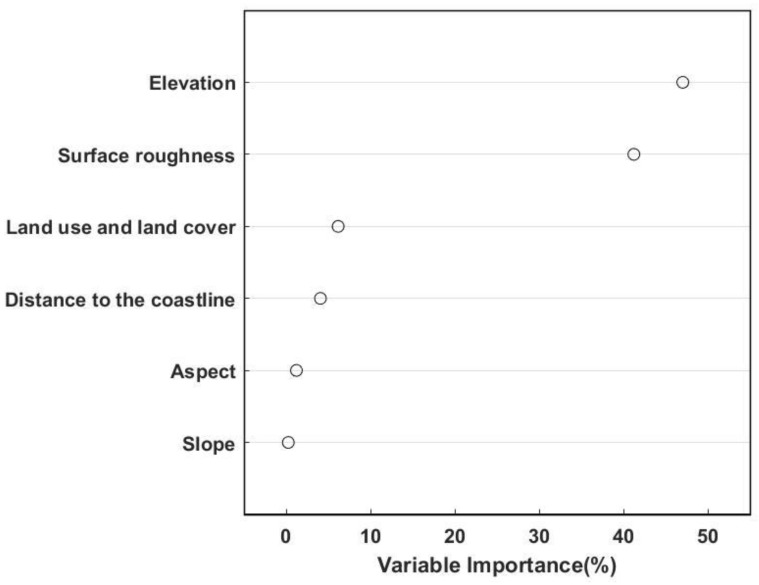
Variable importance plot.

The variable importance test results of the six explanatory variables used in the GWR model are shown in Figure 5. Here, the independent variable and dependent variable are standardized at the same time, which eliminates the influence of dimension and magnitude difference, and makes different variables comparable. We use the absolute value of the standardized regression coefficient to compare the effect of different independent variables on the dependent variable. The results show that all variables used to construct the GWR model contributed to the modeling process. Among them, the elevation and the surface roughness datasets have a high percentage (0.3~0.5), which are the most important independent variables and have a strong impact on the model predictions. Second, the sum of the percentages of land use and land cover and distance to the coastline data sets exceeds 0.1, indicating that these two variables also have a relatively large impact on the model predictions. This is followed by aspect and slope. Based on the above significance results, we included all variables in the precipitation fusion data product.

#### 3.1.2. Impact of Spatial Weighting Functions

**Figure 6:**
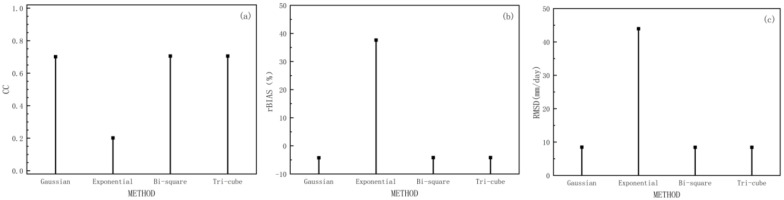
Cross-validation continuous statistics (**a**) CC, (**b**) rBIAS, and (**c**) RMSD on daily combined precipitation data using four weighting functions (Gaussian, exponential, bi-square, and tri-cube).

As mentioned above, commonly used kernel functions include the Gaussian function, exponential function, bi-square function, and tri-cube function, and the corresponding model statistical results are shown in Figure 6. Regarding the use of different weighting functions in the equations, Figure 6b shows that the exponential function leads to the largest rBIAS, and the Gaussian, bi-square, and tri-cube functions all have better performance in terms of bias metrics. Figure 6c shows the RMSD values of the four weighting functions, as well as the exponential function results with larger root mean square difference values, while the other three weighting functions have comparable results. These results confirm that Gaussian, bi-square, and tri-cube functions all have good performance. Regarding the CC indicator, the correlation coefficient values in Figure 6a also corroborate this result.

**Figure 7:**
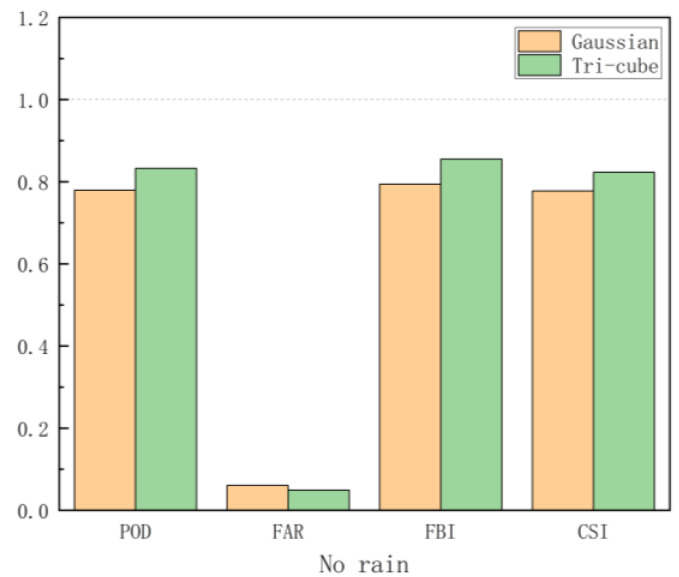
Cross-validation categorical statistics (POD, FAR, FBI, and CSI) for daily combined precipitation data using two weighting functions (Gaussian and tri-cube) in the no-rain precipitation intensity class.

To select the most suitable kernel function under specific model and data conditions, this study compares the categorical indices of the Gaussian function, which performs better than the exponential function in the continuous indices of the continuous kernel function, and the tri-cube function, which performs better than the bi-square function in the continuous indices of the truncated kernel function. In this paper, the precipitation intensity is divided into five categories: no rain ([0,1) mm/d), light rain ([1,5) mm/d), moderate rain ([5,20) mm/d), heavy rain ([20,40) mm/d), and violent rain (≥40 mm/d)). Taking the case of no rain as an example (Figure 7), the POD and CSI results in the no-rain precipitation intensity class ([0,1) mm/d) of the tri-cube function are higher than the Gaussian function, while the tri-cube function is lower than the Gaussian function in terms of the FAR indicator. Overall, the tri-cube function performs the best; thus, the tri-cube function is chosen as the weighting function in this paper.

#### 3.1.3. Impact of Spatial Resolution of Variates

**Figure 8:**
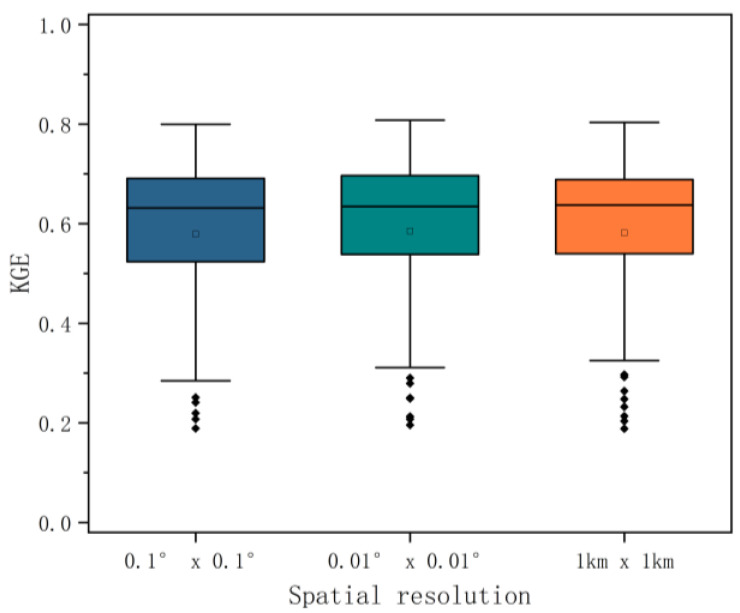
KGE values for the fused precipitation product calculated at three spatial resolutions (0.1°, 0.01°, and 1 km).

Figure 8 plots the KGE values of the merging model under different spatial resolutions. The solid line in a box in Figure 8 represents the median, the edges of the box represent the first and third quartiles, and the whiskers extend to the most extreme data point, which does not exceed 1.5 times of the interquartile range of the box. The open dots are the mean, and the remaining solid dots are outlier data points. The results show that, when the MSWEP source precipitation field with a spatial resolution of 0.1° × 0.1° (~9.2 km × 10.8 km) is resampled to 0.01° × 0.01° (~0.9 km × 1.0 km) or at a resolution of 1 km by 1 km, the effect on the performance of the final merging precipitation products can be ignored. Finally, the precipitation product with a spatial resolution of 1 km by 1 km, which is consistent with DEM and LULC data’s resolution, is generated by merging multi-source precipitation data based on the geographically weighted regression method in this study.

### 3.2. Performance of the Merged Precipitation Product

**Figure 9:**
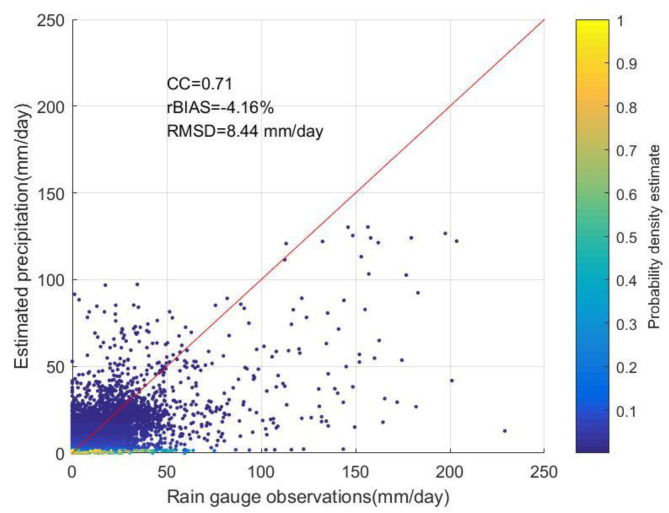
Scatter plots with color density showing rain gauge observation and the estimated precipitation by the GWR model for fusion in 2015.

Figure 9 shows the scatter plot colored by density for the rain gauge observation and estimated precipitation by the GWR-based two-step merging scheme in 2015. The total number of data pairs for model building and the comparison is 25,550. As shown in Figure 9, the GWR model estimates that the precipitation is in good agreement with the rainfall gauge observation, with a CC of 0.71, rBIAS of −4.16%, and RMSD of 8.44 mm/day. Compared with the source precipitation field, these three indicators have been greatly improved. This phenomenon shows that the GWR-based two-step merging scheme performs well in spatial precipitation estimation.

#### 3.2.1. Temporal Assessment of the Merged Product

**Figure 10:**
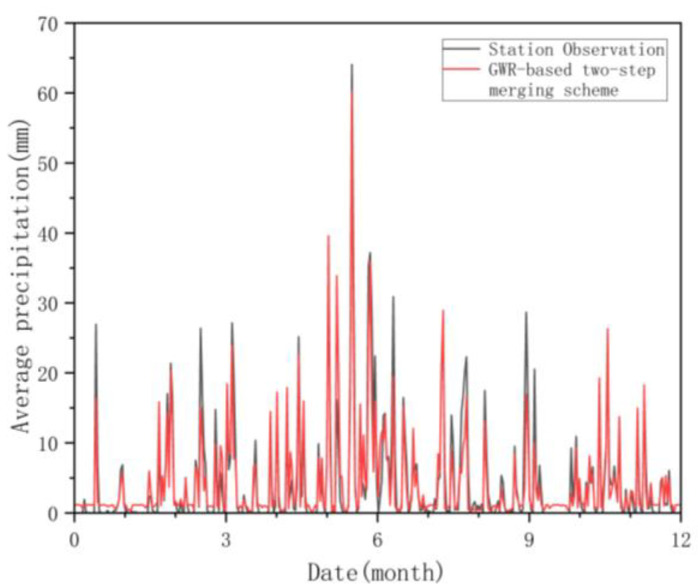
Time series of areal-average daily precipitation of gauge observation and estimated precipitation for 2015.

Figure 10 compares the daily average precipitation of the rain gauges in 2015 with the corresponding model-estimated daily average precipitation. It can be seen from Figure 10 that the model predicts the rainfall trend well. In addition, the model also captures the peak rainfall timing well. These results confirm the effectiveness of the GWR fusion method and its ability to generate reliable precipitation products.

**Figure 11:**
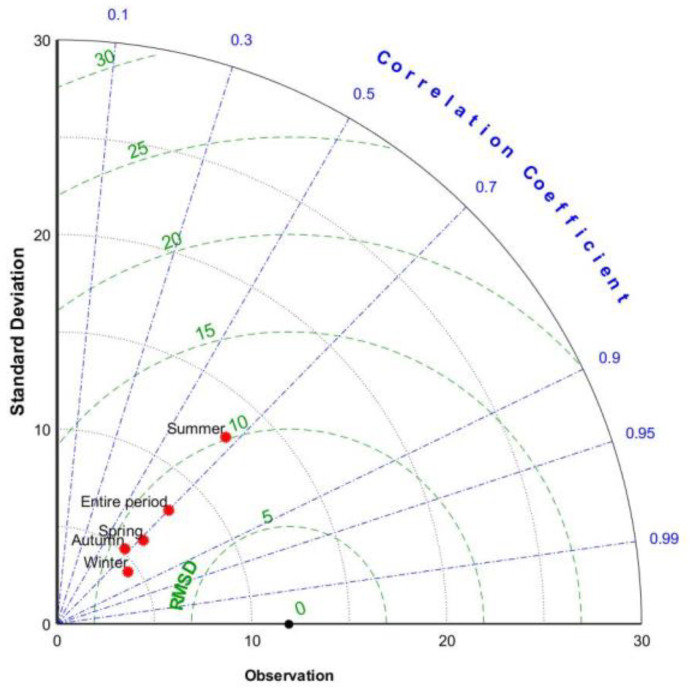
Taylor diagrams for the daily precipitation of the gauge observation and the GWR model across the entire period and different seasons in 2015.

Figure 11 shows the Taylor diagram of daily precipitation of the merged model in 2015 and throughout different seasons to intuitively compare the accuracy of the fusion model in each season. In a Taylor plot, the fusion model is considered a better product if its point is closer to the observed rainfall point and worse if it is not. It is obvious that the maximum deviation of the GWR model precipitation field occurs in the summer, and conversely, it is the lowest in the winter. Spring and autumn have medium performances.

#### 3.2.2. Spatial Assessment of the Merged Product

**Figure 12:**
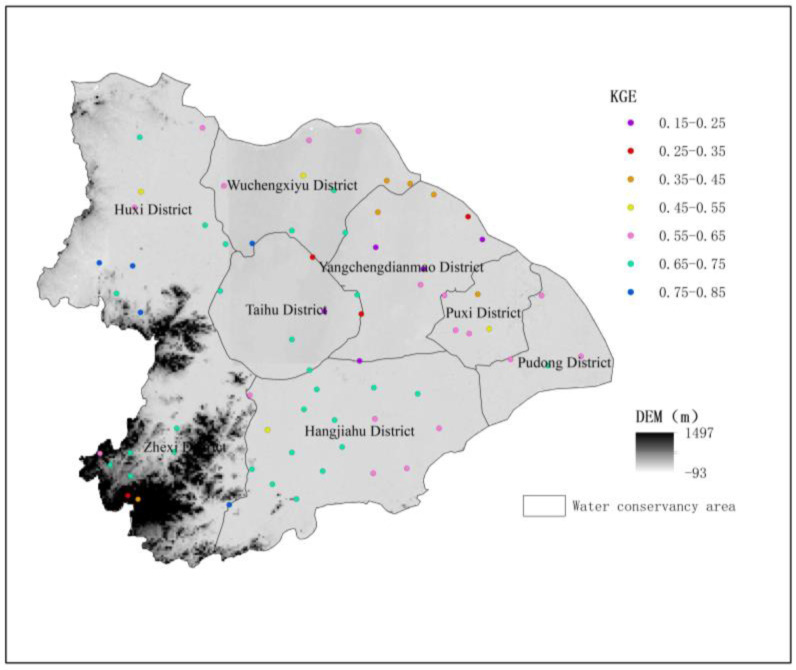
KGE values for the daily precipitation of the GWR model at 70 gauge sites in 2015.

Figure 12 shows the KGE values of predicted precipitation for 70 rainfall stations of the merged model in 2015. KGE values range from 0.1 to 0.9, depending on the space. High KGE values occur in Hangjiahu District, Zhexi District, and Huxi District. The lowest KGE value is distributed in Yangchengdianmao District.

#### 3.2.3. Assessment of Precipitation Intensities

**Figure 13:**
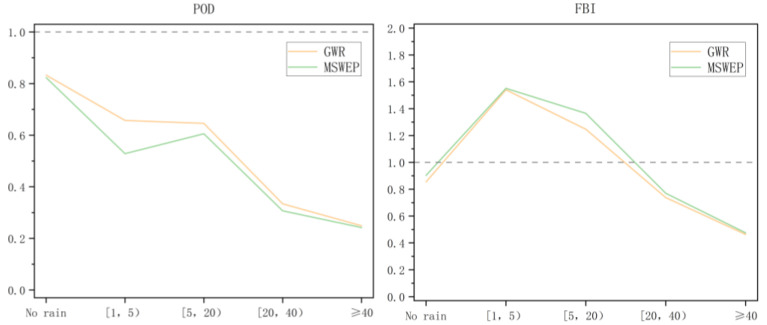

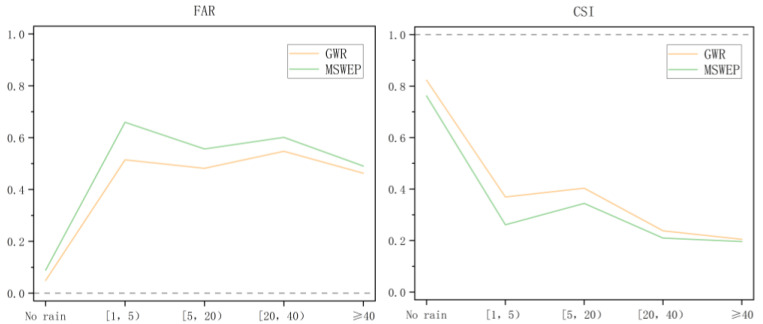
Evaluation statistics for the categorical indices at five precipitation intensity classes (mm/d). From left to right and top to bottom: POD, FAR, FBI, and CSI. The dashed black line represents the optimal value of each index.

Figure 13 plots the values of four categorical indices of five types of precipitation intensity, including no rain ([0,1) mm/d), light rain ([1,5) mm/d), moderate rain ([5,20) mm/d), heavy rain ([20,40) mm/d), and violent rain (≥40 mm/d). It can be seen that both the MSWEP source precipitation field and the GWR fusion precipitation can identify the no-rain event well. In addition, in terms of other precipitation intensities, except for no rain, compared with the MSWEP source precipitation field, the values of the POD and CSI of the GWR model increased, while the FAR decreased, and the FBI was closer to 1. The GWR model precipitation product performs better in the four categorical indices.

## 4. Discussion

In the GWR model used in this paper, the effects of six variables on rainfall in the Taihu Lake Basin are ranked in descending order of elevation, surface roughness, land use and land cover, distance to the coastline, aspect, and slope. In addition to natural factors such as elevation and distance to the coastline, with the rapid advancement of economic development, urbanization, and population increase, the land-use pattern of the Taihu Lake Basin has undergone great changes. At the same time, this pattern has further affected the climate and hydrological environment. This has led to problems such as urban rainstorms and waterlogging. We know that rainfall influences surface roughness. This paper finds that surface roughness also affects rainfall at the same time. This may be related to the land cover. For example, the surface roughness of water bodies and grasslands is different from that of urban areas, and the water storage capacity of the three is different, which affects the water vapor above and, thus, precipitation. Additionally, the Taihu Lake Basin is dominated by plains and is relatively flat, so that the slope and aspect have little influence on the precipitation in this area.

Based on the evaluation of the merging precipitation products in different seasons and different rainfall intensities events, it can be seen that the fusion precipitation product has the best performance in the winter and the worst performance in the summer, because the Taihu Lake Basin has less winter rainfall and strong summer rainfall. Fusion precipitation is significantly worse in capturing heavier precipitation, and there is an underestimation phenomenon. Finding the determinants of underestimation and realizing the correction is the direction of further efforts. In terms of the integrated precipitation assessment of different water conservancy zones, it can be seen that the simulation results are better in areas with large elevation fluctuations, such as Zhexi District, Huxi District, and Hangjiahu District. The relatively flat Yangchengdianmao District has a poor simulation accuracy but may prove the correctness of using elevation to characterize rainfall from another perspective.

In addition, the method in this paper only uses the precipitation measured by a small number of rainfall stations to achieve correction, which effectively improves the accuracy of the final precipitation product. As a next step, we may consider applying this method to no-data or little-data regions. Most of the areas with no or little data have only a few or no rainfall stations due to limited conditions. Compared with the actual measurement of rainfall stations, satellite remote sensing detection information is easier to obtain, and data such as elevation and land use and land cover are also easier to obtain. These are all conditions for considering the application of the GWR method to areas with little precipitation data.

Although the above results indicate the effectiveness of the method adopted in this study, the quality of merging precipitation depends on many factors and can be further improved in the next study. One is the density of surface rainfall stations. Increasing the number of rainfall stations used in the correction may have a positive effect on improving the accuracy of the generated model. The second is the accuracy of satellite source data. Different satellite source data can be selected, or multiple satellite source data can be selected as model input. The third factor is the choice of the resampling method for the satellite-source precipitation field, which may also affect the final results. The fourth is choosing the best response variables and explanatory variables, such as eliminating variables with collinearity and causing heteroscedasticity in the model or choosing different data types as independent variables and dependent variables. Fifth, more weighting methods can be developed or adopted. Only the most common weighting methods are selected in this paper. The sixth factor is reselecting the method for determining the spatial bandwidth, such as determining the bandwidth according to the Akaike information criterion. The seventh factor is extending the GWR method, such as the geographically and temporally weighted regression (GTWR) model, taking the time into account; a multiscale geographically weighted regression (MGWR) model, taking the scale into account; and multiscale geographically and temporally weighted regression (MGTWR) model, taking the time, space, and scale into account simultaneously.

## 5. Conclusions

In this study, considering the geographical and human activities factors, the bilinear interpolation method was successfully used to achieve downscaling, and the GWR technology and the tri-cube function weighting method were used to complete the fusion of precipitation. This significantly improved the spatial resolution and the accuracy of precipitation products in the Taihu Lake Basin. The main findings of this study are as follows.

(1) The variable importance of six explanatory variables selected in this paper is ranked in the order of elevation, surface roughness, land use and land cover, distance to the coastline, aspect, and slope. Elevation has the most significant effect on precipitation distribution. In addition to common geographical elements, land use and land cover directly affect by human activities also have a greater impact on precipitation.
(2) Among the four weighting functions—the Gaussian function, exponential function, bi-square function, and tri-cube function—the tri-cube function has the best performance.
(3) The influence of three different spatial resolutions of the MSWEP source precipitation fields on the performance of the final merged precipitation products can be ignored, so 1 km, which is the best resolution, is selected in this paper.
(4) The maximum deviation of precipitation in the GWR-based two-step merging model occurs in the summer, and conversely, it is the best in the winter. Spring and autumn have medium performances.
(5) The accuracy of the precipitation predicted by the fusion model varies from space to space. Areas with large elevation fluctuations have better simulation results, while areas with relatively flat areas have poor simulation accuracy.
(6) Compared with the MSWEP source precipitation field, the GWR-MSWEP fusion precipitation performs better in the four categorical indices.

The proposed GWR-based two-step merging scheme consisting of downscaling and fusion can successfully achieve and improve the spatial estimation of daily precipitation and obtain precipitation products with higher accuracy and higher spatial resolution in the Taihu Lake Basin.

## Data Availability

The data that support the findings of this study are available from the GloH2O Data Platform (http://www.gloh2o.org/mswep/ (accessed on 24 June 2022)), the United States Geological Survey (https://earthexplorer.usgs.gov/ (accessed on 24 June 2022)), and the Resource and Environmental Data Cloud Platform (https://www.resdc.cn/ (accessed on 24 June 2022)).
